# Prevalence of Ocular, Respiratory and Cutaneous Symptoms in Indoor Swimming Pool Workers and Exposure to Disinfection By-Products (DBPs)

**DOI:** 10.3390/ijerph7041379

**Published:** 2010-03-29

**Authors:** Guglielmina Fantuzzi, Elena Righi, Guerrino Predieri, Pierluigi Giacobazzi, Katia Mastroianni, Gabriella Aggazzotti

**Affiliations:** University of Modena and Reggio Emilia, Department of Public Health Sciences, Via G. Campi, 287, 41121 Modena, Italy, E-Mails: elena.righi@unimore.it (E.R.); guerrino.predieri@unimore.it (G.P.); pierluigi.giacobazzi@unimore.it (P.G.); 35100@studenti.unimore.it (K.M.); gabriella.aggazzotti@unimore.it (G.A.)

**Keywords:** respiratory, ocular and cutaneous symptoms, irritative symptoms, occupational exposure, swimming pools, trihalomethanes

## Abstract

The objective of this cross-sectional study was to investigate the prevalence of self-reported respiratory, ocular and cutaneous symptoms in subjects working at indoor swimming pools and to assess the relationship between frequency of declared symptoms and occupational exposure to disinfection by-products (DBPs). Twenty indoor swimming pools in the Emilia Romagna region of Italy were included in the study. Information about the health status of 133 employees was collected using a self-administered questionnaire. Subjects working at swimming pools claimed to frequently experience the following symptoms: cold (65.4%), sneezing (52.6%), red eyes (48.9%) and itchy eyes (44.4%). Only 7.5% claimed to suffer from asthma. Red eyes, runny nose, voice loss and cold symptoms were declared more frequently by pool attendants (lifeguards and trainers) when compared with employees working in other areas of the facility (office, cafe, *etc.*). Pool attendants experienced generally more verrucas, mycosis, eczema and rash than others workers; however, only the difference in the frequency of self-declared mycosis was statistically significant (p = 0.010). Exposure to DBPs was evaluated using both environmental and biological monitoring. Trihalomethanes (THMs), the main DBPs, were evaluated in alveolar air samples collected from subjects. Swimming pool workers experienced different THM exposure levels: lifeguards and trainers showed the highest mean values of THMs in alveolar air samples (28.5 ± 20.2 μg/m^3^), while subjects working in cafe areas (17.6 ± 12.1 μg/m^3^), offices (14.4 ± 12.0 μg/m^3^) and engine rooms (13.6 ± 4.4 μg/m^3^) showed lower exposure levels. Employees with THM alveolar air values higher than 21 μg/m^3^ (median value) experienced higher risks for red eyes (OR 6.2; 95% CI 2.6–14.9), itchy eyes (OR 3.5; 95% CI 1.5–8.0), dyspnea/asthma (OR 5.1; 95% CI 1.0–27.2) and blocked nose (OR 2.2; 95% CI 1.0–4.7) than subjects with less exposure. This study confirms that lifeguards and trainers are more at risk for respiratory and ocular irritative symptoms and cutaneous diseases than subjects with other occupations at swimming pool facilities.

## 1. Introduction

There is growing concern about the onset of asthmatic conditions and other respiratory ailments in subjects attending indoor swimming pools. Recent epidemiological studies report unusually high prevalence rates of ocular and respiratory symptoms as well as asthmatic conditions—both in swimmers (mainly children) and in occupationally exposed subjects—as a consequence of their environmental exposure to chemical derivatives of pool water disinfection treatments [1–6]. Moreover, in subjects working at indoor swimming pools, such as hydro-therapists, recent evidence has shown a high frequency of skin diseases such as contact dermatitis [7].

It is well known that pool water chlorination can produce halogenated disinfection by-products (DBPs), most of which are potentially harmful to human health. People working at indoor swimming pools are regularly exposed to DBPs at concentrations that can vary according to chemical and physical factors such as type of source water, disinfecting agents, water temperature, water pH, water replacement rate, ventilation rate and the number of swimmers [8].

Some volatile halogenated compounds can be found in swimming pool atmospheres as a consequence of the chemical reactions between chlorine, ammonia and amino-compounds from the sweat and urine of swimmers [8,9]. Chloramines, especially trichloramine, are considered the causal agent for irritated eye, nose and throat symptoms, as well as asthma, in swimmers and subjects working at indoor swimming pools [1,2,4,5,9,10]. However, in a recent review of childhood asthma and various environmental exposures at swimming pools, the authors claim that exposure to trichloramine has not yet been adequately characterized to clearly demonstrate an association with asthma [6]. Furthermore, the analytical method commonly used to detect trichloramine in the atmosphere is not considered specific enough to differentiate between inorganic and organic chloramines and other volatile DBPs with possible irritative effects [11].

Among DBPs, trihalomethanes (THMs) are the most commonly investigated and are considered representative of total DBPs exposure at swimming pools [8]. As a consequence, work-related DBPs exposure can be evaluated by environmental and biological monitoring of THMs. Because THMs are volatile, they can be found in swimming pool atmospheres depending on their presence in the pool water and the number of swimmers in the pool agitating the water [12–15].

The majority of epidemiological studies address work-related exposure *via* environmental monitoring; however, environmental monitoring does not take into account factors that can influence individual intake of DBPs such as the frequency and type of job activities performed at the swimming pool. In fact, while lifeguards are generally exposed to DBPs only *via* inhalation, trainers who enter the pool water in the course of their work can be exposed to DBPs not only by inhalation, but also by ingestion and dermal contact.

Biological monitoring is a good procedure for investigating occupational exposure; among human biomarkers, alveolar air analysis is considered a simple and non-invasive method for studying workers’ exposure to volatile substances at indoor swimming pools [12,13,16,20]. Previous studies on this topic demonstrated good correlation between THMs measured in breath and corresponding indoor swimming pool environments, which confirms that alveolar air analysis can be used to evaluate the overall intake of DBPs [13,19].

The aim of this study was to investigate the prevalence of self-reported health conditions in subjects working at indoor swimming pools in order to evaluate associations between certain health effects and occupational exposure to DBPs. Exposure was evaluated *via* both environmental and biological monitoring. Analysis of THMs in alveolar air samples was considered representative of total DBPs intake and was used to investigate whether such a biomarker could be useful for determining associations with health effects at indoor swimming pools.

## 2. Material and Methods

In this cross-sectional study, 20 indoor swimming pools in the Emilia-Romagna region of Northern Italy were investigated during October 2007–April 2008. In total, 133 subjects were recruited from among the employees at the monitored swimming pools. Each participant provided informed consent after a brief meeting regarding the purpose and methods of the study. Given the non-invasive character of the study, no ethical approval was required according to Italian law.

A questionnaire was drawn up to record information about health status and working conditions at the swimming pools, as well as personal information about the subjects (age, sex, position at the swimming pool, number of working hours per day and week, previous or similar jobs, smoking habits and medical history). The frequency of self-reported respiratory, ocular and cutaneous symptoms (red eyes, itchy eyes, eye infections, watery eyes, nasal itch, sneezing, cold, runny nose, blocked nose, voice loss, pharyngitis/bronchitis, coughing, wheezing, shortness of breath and dyspnea/asthma) was also recorded. Information about pool water treatment and disinfection procedures was also collected. All sampling sessions were carried out during the winter with closed windows. During each session, three samples of pool water were collected from a depth of 20 cm at three different points near the edge of the pool; the mean value was considered representative of THMs concentration in the water during the sampling session. Environmental air samples were collected at a height of 150 cm above the edge of the pool. Alveolar air samples were collected from only 115 workers: 18 subjects did not agree to biological monitoring [16].

Temperature, pH, combined and free chlorine, and THMs were analyzed in the pool water samples. Water temperature and pH were detected *in situ* with a thermometer and a portable pH meter; free and combined chlorine were evaluated poolside using a colorimetric method based on N,N-diethyl-*p-*phenylendiamine (DPD) [21].

The level of THMs in water samples were analyzed using a static head space gas chromatographic technique (SHS-GC) according to previous studies [12,16]. A Varian 3380 gas chromatograph equipped with a ^63^Ni electron-capture detector (ECD) and a Vocol capillary column (30 m × 0.53 mm I.D., film thickness 3.0 μm (Supelco)). Quantitative and qualitative analyses of THMs were managed using CHROMELEON DIONEX, 6.0 software. Calibration was performed according to the external standard method. Standards of THMs were prepared with pure analysis reagents (Supelco) in specific concentrations of methanol. A working standards solution (concentration 1–8 μg/L) was prepared every day in synthetic water (chloride 100 ppm, nitrate 20 ppm and sulfate 150 ppm). The detection limit for each THMs sample was 0.1 μg/L. Aliquots of 5 ml from the water sample, external standard and control blank (vials with THMs-free water) were each placed into a 10 mL glass vial, sealed, and heated to a specific temperature (1 hour at 37 °C). After incubation, 100 μL of the head-space sample was injected directly into the GC using a gas-tight syringe (Hamilton).

The levels of THMs were investigated for both environmental and alveolar air samples. Ambient air sampling was carried out from different areas of the building: poolside, in the reception area and in the café area. Environmental air samples were collected over a period of 2 h in Tedlar bags (2 l) using a pump. Tedlar bags have a push-pull valve for the sampling phase and a septa port for the analysis phase. The pump flow (Buck Pump, Supelco, USA) was calibrated to 15 cm^3^/min to obtain a complete fill over the course of the sample session. Alveolar air samples were collected in 34 cm^3^ one-way glass tubes with two valves. Subjects were asked to breathe normally into the tube with open valves. At the end of expiration, the valves were closed. For analysis, the tubes were heated to 37 °C to recreate conditions at the time of sampling.

Levels of THMs in ambient and alveolar air were analyzed by direct injection of samples into the VARIAN 3380 chromatograph using a gas-tight syringe (Hamilton). Calibration was performed as mentioned above, and the limit of detection for each THMs sample, both in environmental and alveolar air samples, was 1 μg/m^3^ [12].

### 2.1. Statistical Methods

Statistical analyses were performed using SPSS (Statistical Package for Social Sciences) version 16.0 for Windows. [22] Exposed and non-exposed workers were compared according to ocular, cutaneous, asthmatic and other respiratory symptoms using the X^2^ test with probabilities below 0.05 considered statistically significant. Associations between self-reported health symptoms and occupational THMs exposure were evaluated using a univariate logistic regression adjusted for confounding variables such as sex, age and smoking habits. The odds ratios (ORs) and their 95% confidence intervals (95% CI) were computed.

## 3. Results

Descriptive characteristics of the investigated subjects are reported in Table 1. Most of the swimming pool workers were female (52.6%) with a mean age of 33 years, non-smoking (58.7%), and had worked in swimming pool facilities for an average of eight years. No statistical differences were observed between males and females. Employees had mean weekly work totals of 25.2 ± 14.1 hours, corresponding to 4.7 ± 2.4 hours/day. According to the questionnaire data, 50% of subjects had attended indoor swimming pools for more than 20 years, mainly as swimmers.

Regarding irritative symptoms, the general prevalence of ocular, respiratory and cutaneous symptoms reported in the questionnaire data is shown in Table 2. In the same table, the prevalence data are shown by major job activities: lifeguards and trainers were compared with subjects who had other activities at the swimming pool facilities such as managers, receptionists, technicians and bartenders.

Among self-reported ocular symptoms, employees declared frequently having red eyes (48.9%) and itchy eyes (44.4%). Red eyes, itchy eyes and eye infections were more frequent among lifeguards and trainers than other employees, even if only red eyes showed a statistical difference in frequency of occurrence (p < 0.05).

Regarding asthma and other respiratory symptoms, cold and sneezing were the most frequent self-declared symptoms (65.4% and 52.6%, respectively) while only 7.5% of the total employees reported suffering from an asthmatic condition. No difference was observed in the prevalence of self-reported asthmatic symptoms between lifeguards and trainers and other employees, while statistically significant differences were observed in cold occurrence, runny nose and voice loss, which were reported more frequently by pool attendants (lifeguards and trainers).

With regard to self-reported symptoms of cutaneous diseases, the frequencies of verrucas, mycosis, eczema and rash are also reported in Table 2. Lifeguards and trainers experienced generally more verrucas, mycosis, eczema and rash than others workers at swimming pools. The difference in the frequency of self-declared mycosis was statistically significant (p < 0.05). Moreover, no difference in skin symptoms was observed in trainers who entered pool waters during their working activities when compared with employees without any regular pool water contact.

With regard to physical and chemical parameters, water temperature showed values ranging from 27 °C to 29 °C (28 °C ± 1 °C), and a mean pH of 7.3 ± 0.2 was measured. According to the disinfection practices, 11 swimming pools (55%) were disinfected with both hypochlorite and chloroisocyanurate compounds, four were treated with only hypochlorite compounds and five with only chloroisocyanurate compounds. Data on free and combined chlorine in water ranged from 0.7 to 2.0 mg/L and from 0.1 to 0.9 mg/L, respectively. A mean value of combined chlorine was found (0.5 ± 0.2 mg/L). Only five indoor swimming pools (25%) showed a combined chlorine value lower than 0.4 mg/l, which is the lowest permissible value by Italian law. The levels of THMs in pool water ranged from 7 μg/L to 134 μg/L with a mean value of 41.4 ± 30.0 μg/L and a median value of 28.5 μg/L.

In Table 3, THMs levels in ambient air samples are reported according to the different working areas of the swimming pool buildings, particularly the poolside area, the reception and the café area. In the same table, THMs concentrations in alveolar air samples from 115 investigated workers are shown. The mean value of THMs from ambient air samples collected at poolside was 81.1 ± 45.5 μg/m^3^ while lower THMs mean values were observed in the café areas (34.7 ± 29.3 μg/m^3^) and in the reception areas (30.0 ± 29.9 μg/m^3^); the differences were statistically significant (p < 0.05).

Regarding occupational exposure, lifeguards and trainers showed the highest mean value of THMs in alveolar air samples, while subjects working in the café areas, offices or engine rooms showed lower exposure levels. A total THMs mean value of 23.4 ±18.3 μg/m^3^ (median value = 21 μg/m^3^) was observed in alveolar air samples collected from all investigated employees (115).

THMs in alveolar air and in ambient air samples were highly correlated (r = 0.672; p < 0.001).

Taking into account the median values of THMs in alveolar air samples, two occupational exposure levels were considered: a low level of exposure corresponding to the median THMs alveolar air value in receptionists, managers and technicians (12 μg/m^3^), and a high level of exposure corresponding to the median value of THMs in alveolar air samples in all investigated subjects (21 μg/m^3^).

In Table 4, associations between self-reported health symptoms and occupational THMs exposure, evaluated according to level of exposure, are reported. A logistic regression model was applied with confounding variables of sex, age and smoking habits; data are expressed as odds ratios and their confidence intervals. Referent groups are subjects with alveolar air values ≤12 μg/m^3^ and ≤21 μg/m^3^, respectively. Subjects with THMs levels in alveolar air higher than 12 μg/m^3^ showed an increased risk for red eyes (adjusted OR 4.3; 95% CI 1.8–10.3) and itchy eyes (adjusted OR 3.5; 95% CI 1.5–8.0) when compared with subjects having lower levels of THMs in their alveolar air samples. When a cut-off value of 21 μg/m^3^ was taken into account, a higher risk was observed for red eyes (adjusted OR 6.2; 95% CI 2.6–14.9), while no incremental risk was observed for itchy eyes (adjusted OR 3.5; 95% CI 1.5–8.5).

Only 10 subjects out of the 115 who participated in biological monitoring reported an asthmatic condition. Of these, eight had concentrations of THMs in their alveolar air samples higher than 21 μg/m^3^; thus the adjusted OR was 5.1 (95% CI 1.0–27.2). Regarding other respiratory symptoms, only blocked nose was more frequent (adjusted OR 2.2; 95% CI 1.0–4.7) in subjects with THMs levels in alveolar air higher than 21 μg/m^3^.

## 4. Discussion

This cross-sectional study was performed to evaluate the prevalence of ocular, respiratory and cutaneous symptoms in subjects occupationally exposed to DBPs. Higher prevalence rates for irritative ocular and respiratory symptoms were experienced by indoor swimming pool workers with jobs as lifeguards and trainers than those with other working activities such as managers, receptionists, technicians and bartenders. In particular, red eyes, cold symptoms, runny nose and voice loss were declared more frequently by lifeguards and trainers when compared with employees working in other areas of the swimming pool facilities (office, cafe, *etc.*).

High prevalence of irritative symptoms and other health endpoints in people attending indoor swimming pools are consistent with prior research. Epidemiological studies have linked irritative symptoms to DBPs, and in particular to trichloramine, which is generally considered the most important risk factor for irritative symptoms in swimmers and occupationally exposed subjects at indoor swimming pools [1,5,25]. However, some authors suggest that there may be other volatile DBPs with potential irritant properties in indoor pool environments [11,26].

Recent epidemiological studies have raised questions about whether inhalation of DBPs may cause or exacerbate existing asthma, but the results of the studies are still inconclusive. Few epidemiological studies have assessed this association in occupationally exposed subjects [1,2,5,27–32]. In a recent review on childhood asthma and environmental exposure at swimming pools, some methodological gaps were shown among epidemiological studies related to asthma characterisations and DBPs exposure assessment.

In this study, DBPs exposure assessment was evaluated both by environmental and biological monitoring of THMs, which represent the main DBPs from water disinfection. At the investigated swimming pools, environmental levels of THMs generally appeared low and in agreement with prior literature [13–15,20]. However, airborne THMs values at poolside were higher than values in café and reception areas and, as a consequence, swimming pool employees showed differing occupational exposures; the highest exposure levels (according to THMs analysis from alveolar air) were measured in poolside trainers or lifeguards who inhaled THMs directly from the pool water. This result confirms that different environmental exposures in the swimming pool environment can induce different internal doses in occupationally exposed subjects. Moreover, the correlation between ambient and alveolar air samples confirms that breath analysis can be considered a suitable biomarker for occupational exposure to volatile compounds such as THMs [13,19].

Asthma-related conditions, evidenced in subjects with higher level of THMs in alveolar air samples when compared with subjects with low levels of THMs exposure, suggest the potential use of this biomarker to identify exposure conditions that can trigger to irritative symptoms linked to swimming pools attendance.

Regarding cutaneous diseases, a general increase in cutaneous symptoms was observed in pool attendants when compared with other swimming pool employees, with a significantly higher frequency of mycosis. An increased risk for contact dermatitis has been shown in hydro-therapists who reported skin diseases after the beginning of their work at the pool [7,24]. The authors also found contact dermatitis to be more frequent in subjects working at swimming pools where pool waters were treated with gaseous chlorine. Even though our study does not specifically include hydro-therapists, we did not find more eczema or rash in trainers who entered the pool water during their teaching activities when compared with other investigated employees.

This study presents some limitations. First, information about health status was collected using a self-administered questionnaire, with no clinical diagnosis or medical control. As a consequence, recall bias may affect the data. Another critical aspect of this study deals with the diagnosis of asthma. Asthma was defined by asking if the subject had ever been diagnosed with asthma by a physician (*i.e.*, doctor-diagnosed asthma); thus, misclassifications may have occurred. Moreover, asthma was declared only by 10 of the 133 investigated subjects, which makes the observed risk statistically weak.

Our study showed some notable results. All employees working during our visit to each swimming pool agreed to participate in the study; thus, there is no selection bias. We tried to obtain information about the health status of workers who were not present at the swimming pool during the sampling session, but only a few questionnaires came back, which were not considered. In this study, DBPs exposure in subjects working in indoor swimming pools was evaluated by alveolar air analysis and so exposure was assessed at the individual level. Alveolar air analysis is a safe and non-invasive technique, and is therefore usually more acceptable to employees, resulting in higher levels of participation in exposure studies.

## 5. Conclusion

The present study shows that irritative ocular and respiratory symptoms are more frequent in lifeguards and trainers than in subjects with other work activities at swimming pool facilities such as managers, receptionists, technicians and bartenders. Moreover, the observed association between asthma and occupational exposure, as evaluated by THMs in alveolar air, seems to confirm that subjects working at swimming pools may be at risk for asthma, especially those working poolside. Future work should attempt to confirm these findings in a larger group of occupationally exposed subjects.

## Figures and Tables

**Table 1.** Descriptive characteristics of the 133 investigated subjects working at the 20 indoor swimming pools.

Variables	n.	%
Gender		
Male	63	47.4
Female	70	52.6

Age (years)		
<30	42	32.3
30–39	55	42.3
≥40	33	25.4

Smoking habits		
Smoker	55	41.4
Ex-smoker	13	9.8
Never smoked	65	48.9

Duration of occupational activity at indoor swimming pools (years)		
1–5	56	42.1
6–10	43	32.3
>10	34	25.6

Job		
Lifeguard/trainer	82	61.7
Bartender	9	6.8
Receptionist/pay desk	22	16.5
Manager/office worker	15	11.3
Technician	5	3.8

**Table 2.** Prevalence of ocular, cutaneous, asthma-related and other respiratory symptoms in the 133 occupationally exposed subjects.

SYMPTOMS	Total employees (133)		Lifeguards and trainers (82)		Other employees (51)		OR (95% CI) p value	Adjusted OR (95% CI) p value
	n	%	n	%	n	%		
OCULAR SYMPTOMS								
Red eyes	65	48.9	48	58.5	17	33.3	2.8 (1.4–5.9) 0.005*	2.9 (1.3–6.1) 0.006*
Itchy eyes	59	44.4	41	50.0	18	35.3	1.8 (0.9–3.8) 0.09	1.9 (0.9–4.0) 0.08
Eye infection	13	9.8	11	13.6	2	4.0	3.8 (0.8–17.8) 0.09	4.0 (0.8–19.2) 0.08
Watery eyes	32	24.1	20	24.4	12	23.5	1.0 (0.5–2.4) 0.91	1.1 (0.5–2.4) 0.89

ASTHMA RELATED SYMPTOMS								
Coughing	51	38.3	32	62.7	19	37.3	1.1 (0.5–2.2) 0.84	1.1 (0.5–2.4) 0.75
Wheezing	23	17.3	12	14.6	11	21.6	0.6 (0.3–1.5) 0.31	0.6 (0.2–1.5) 0.28
Shortness of breath	13	9.8	8	9.8	5	9.8	1.0 (0.3–3.2) 0.99	1.0 (0.3–3.2) 0.97
Asthma	10	7.5	7	8.5	3	5.9	1.5 (0.4–6.1) 0.58	1.6 (0.4–6.7) 0.54

OTHER RESPIRATORY SYMPTOMS								
Nasal itch	47	35.3	31	37.8	16	31.4	1.3 (0.6–2.8) 0.45	1.5 (0.7–3.3) 0.28
Sneezing	70	52.6	45	54.9	25	49.0	1.3 (0.6–2.5) 0.51	1.2 (0.6–2.5) 0.57
Cold	87	65.4	59	72.0	28	54.9	2.1 (1.0–4.4) 0.05*	2.1 (1.0–4.4) 0.05*
Runny nose	42	31.6	32	39.0	10	19.6	2.6 (1.2–6.0) 0.02*	2.6 (1.1–6.0) 0.02*
Blocked nose	62	46.6	43	52.4	19	37.3	1.9 (0.9–3.8) 0.09	1.8 (0.9–3.7) 0.12
Voice loss	57	42.9	43	52.4	14	27.5	2.9 (1.4–6.2) 0.005*	3.3 (1.5–7.2) 0.003*
Bronchitis	29	21.8	16	19.5	13	25.5	0.7 (0.3–1.6) 0.42	0.7 (0.3–1.7) 0.43

CUTANEOUS SYMPTOMS								
Verrucas	22	16.5	17	20.7	5	9.8	2.4 (0.8–7.0) 0.11	2.9 (0.9–8.7) 0.06
Mycosis	21	15.8	18	22.0	3	5.9	4.5 (1.3–16.2) 0.02*	4.6 (1.3–16.7) 0.02*
Eczema	12	9.0	8	9.8	4	7.8	1.3 (0.4–4.5) 0.71	1.4 (0.4–5.0) 0.62
Rash	27	20.3	18	22.0	9	17.6	1.3 (0.5–3.2) 0.55	1.4 (0.6–3.5) 0.47

Data are presented as odds ratios (95% confidence interval) and are adjusted for age, sex and smoking habits.

* p < 0.05.

**Table 3.** THMs concentrations in ambient air samples collected from different working areas at indoor swimming pool facilities and THMs values in alveolar air samples from the investigated employees.

Working areas (n)	THMs in ambient air μg/m^3^		Employees (n)	THMs in alveolar air μg/m^3^	

	Mean ± SD	Median		Mean ± SD	Median
Poolside (20)	81.1 ± 45.5	76	Lifeguards and trainers (72)	28.5 ± 20.2	24
Cafe (8)	34.7 ± 29.3	31	Bartenders (9)	17.6 ± 12.1	11
Reception (18)	30.0 ± 29.9	28	Receptionists/Office/Managers/Technicians (34)	14.7 ± 10.4	12
			
			Total employees (115)	23.5 ±18.3	21

**Table 4.** Self-reported symptoms and THMs levels from alveolar air samples for 115 occupationally exposed subjects.

Symptoms	THMs alveolar air >12 μg/m^3^*** Adjusted OR (95% CI)	THMs alveolar air >21 μg/m^3^*** Adjusted OR (95% CI)
IRRITATIVE SYMPTOMS		
Nasal itch	1.2 (0.5–2.8)	0.9 (0.4–2.0)
Sneezing	1.0 (0.5–2.3)	0.9 (0.4–1.9)
Red eyes	4.3 (1.8–10.3)*	6.2 (2.6–14.9)**
Itchy eyes	3.5 (1.5–8.5)*	3.5 (1.5–8.0)*

ASTHMA-RELATED SYMPTOMS		
Coughing	2.0 (0.8–4.8)	1.4 (0.6–3.0)
Wheezing	1.8 (0.6–5.6)	1.2 (0.5–3.4)
Shortness of breath	2.4 (0.5–11.9)	1.0 (0.3–3.7)
Dyspnea/asthma	6.5 (0.7–56.6)	5.1 (1.0–27.2)*

OTHER OCULAR AND RESPIRATORY SYMPTOMS		
Cold	1.5 (0.7–3.4)	1.3 (0.6–2.8)
Runny nose	1.4 (0.6–3.4)	1.7 (0.7–3.8)
Blocked nose	2.0 (0.9–4.6)	2.2 (1.0–4.7)*
Voice loss	1.0 (0.4–2.2)	1.5 (0.7–3.2)
Pharyngitis/bronchitis	1.0 (0.3–1.7)	0.7 (0.3–2.1)
Eye infections	6.3 (0.8–51.7)	2.8 (0.7–11.5)
Watery eyes	1.5 (0.6–3.9)	1.7 (0.7–4.2)

Data are presented as odds ratio (95% confidence interval), adjusted for age, sex and smoking habits.

* p < 0.05;

** p< 0.001

*** Referent groups are subjects with alveolar air values ≤ 12 μg/m^3^ and ≤ 21 μg/m^3^, respectively.
